# Impact of Electronic Cigarettes, Heated Tobacco Products and Conventional Cigarettes on the Generation of Oxidative Stress and Genetic and Epigenetic Lesions in Human Bronchial Epithelial BEAS-2B Cells

**DOI:** 10.3390/toxics11100847

**Published:** 2023-10-10

**Authors:** Gianni Zarcone, Marie Lenski, Thomas Martinez, Smaïl Talahari, Ophélie Simonin, Guillaume Garçon, Delphine Allorge, Fabrice Nesslany, Jean-Marc Lo-Guidice, Anne Platel, Sébastien Anthérieu

**Affiliations:** Univ. Lille, CHU Lille, Institut Pasteur de Lille, ULR 4483, IMPECS-IMPact de l’Environnement Chimique sur la Santé, F-59000 Lille, France; gianni.zarcone@univ-lille.fr (G.Z.); marie.lenski@chu-lille.fr (M.L.); smail.talahari@pasteur-lille.fr (S.T.); ophelie.simonin@univ-lille.fr (O.S.); guillaume.garcon@univ-lille.fr (G.G.); delphine.allorge@univ-lille.fr (D.A.); fnesslany@erbc-group.com (F.N.); jean-marc.lo-guidice@univ-lille.fr (J.-M.L.-G.); anne.platel@pasteur-lille.fr (A.P.)

**Keywords:** heat-not-burn tobacco, e-cigarette, lung, cytotoxicity, genotoxicity, Nrf2

## Abstract

Electronic cigarettes (e-cig) and heated tobacco products (HTP) are often used as smoking cessation aids, while the harm reduction effects of these alternatives to cigarettes are still the subject of controversial debate, in particular regarding their carcinogenic potential. The objective of this study is to compare the effects of e-cig, HTP and conventional cigarette emissions on the generation of oxidative stress and genetic and epigenetic lesions in human bronchial epithelial BEAS-2B cells. Our results show that HTP were less cytotoxic than conventional cigarettes while e-cig were not substantially cytotoxic in BEAS-2B cells. E-cig had no significant effect on the Nrf2 pathway, whereas HTP and cigarettes increased the binding activity of Nrf2 to antioxidant response elements and the expression of its downstream targets HMOX1 and NQO1. Concordantly, only HTP and cigarettes induced oxidative DNA damage and significantly increased DNA strand breaks and chromosomal aberrations. Neither histone modulations nor global DNA methylation changes were found after acute exposure, regardless of the type of emissions. In conclusion, this study reveals that HTP, unlike e-cig, elicit a biological response very similar to that of cigarettes, but only after a more intensive exposure: both tobacco products induce cytotoxicity, Nrf2-dependent oxidative stress and genetic lesions in human epithelial pulmonary cells. Therefore, the health risk of HTP should not be underestimated and animal studies are required in order to determine the tumorigenic potential of these emerging products.

## 1. Introduction

Smoking is an established risk factor for a variety of diseases, including lung cancer. About 80% of lung cancer deaths are attributable to cigarette smoking and the mix of carcinogens to which bronchial airways are exposed [1]. During cigarette smoking, both the chemicals naturally present in the tobacco and the secondary chemicals generated by pyrolysis are inhaled by the smoker. To date, over 7000 chemicals have been identified in tobacco smoke, including hundreds of chemicals that are toxic and more than 70 that can cause, initiate or promote cancer (formaldehyde, acetaldehyde, benzene, nitrosamines, arsenic, cadmium, etc.) [2]. Smoking cessation is now the only way to eliminate this risk factor. In recent years, new electronic nicotine delivery products (i.e., e-cigarettes (e-cig)) and new tobacco products (i.e., heated tobacco products (HTP)) have emerged, and many smokers use them as an alternative to conventional tobacco cigarettes in order to stop or reduce their cigarette consumption. These devices are generally perceived as less harmful than cigarettes and have rapidly gained popularity in the absence of any real evidence of their safety.

E-cig are battery-powered devices with prefilled cartridges/pods or refillable tanks containing a liquid mixture (called e-liquid) composed of propylene glycol and/or glycerol, nicotine and flavors. The user activates the atomizer’s heating coil by depressing the device’s power button during inhalation [3,4,5,6]. More recently, the tobacco industry launched HTP, devices that heat tobacco and glycerin sticks (called ”heets”) at temperatures less than 350 °C [7]. The tobacco industry claims that these products present fewer health risks than smoking tobacco products [8,9,10]. Tobacco companies, such as Philips Morris International, state that HTP emit, on average, 90% lower levels of harmful chemicals than cigarette smoke (CS) due to the lack of combustion of tobacco [11]. In a previous study, we compared the chemical composition of HTP, e-cig and cigarette emissions and confirmed that HTP emitted lower amounts of carbonyl compounds and polycyclic aromatic hydrocarbons (PAHs) than tobacco cigarettes, but we showed that HTP produced higher amounts than e-cig [12]. Despite the reduction in emissions of harmful compounds compared to cigarettes, a proportional reduction in risk to users of HTP or e-cig cannot be guaranteed. The levels of harmful constituents produced may still be significant and pose a risk to health, in particular the presence of genotoxic carcinogenic chemicals, such as formaldehyde, acetaldehyde and benzo[a]pyrene [12,13]. Due to their DNA interaction properties, these chemicals are considered to have no safe threshold or dose [14]. Therefore, they are expected to cause genotoxic and carcinogenic risks in humans, even at very low concentrations.

E-cig and especially HTP have not yet been used long enough for epidemiological studies to conclude whether or not they are carcinogenic in humans. Indeed, it is known that it usually takes between one and two decades for tobacco smokers to develop lung cancer [15]. Therefore, animal models and in vitro mechanistic studies are necessary as surrogates to study the carcinogenic effects of HTP and e-cig and to better understand the molecular and cellular changes induced by these new products. The development of lung cancer is a complex process, involving a series of genetic and epigenetic alterations such as DNA methylation, histone post-translational modifications and abnormal expression of non-coding RNAs including microRNAs (miRNAs) [16,17]. Smoking directly induces oxidative stress and may activate normally inactive proto-oncogenes, inactivate otherwise active tumor suppressor genes and may induce mutations in DNA repair genes [18]. In addition, exposure to CS has been found to be associated with epigenetic changes in the respiratory tract [19]. Indeed, CS caused global DNA hypomethylation and post-translational modifications of histone residues in human smokers [20,21,22], in the lungs of exposed mice [23,24] and in human bronchial epithelial cells treated with CS extract [25,26,27]. These genetic and epigenetic alterations interact at all stages of cancer development, working together to promote cancer progression [28].

While the genotoxic potential and epigenetic alterations of cigarettes are well known, studies on the genotoxicity of e-cig [3,29,30,31,32,33] and HTP [11,34,35,36,37,38,39] give contradictory conclusions and the epigenetic impacts of these new products are still poorly described in the literature [19,24]. More importantly, there are currently few studies that compare the potential genetic and epigenetic effects of HTP and e-cig emissions, although such a comparison, by independent works, seems essential since they are both alternatives to cigarettes and are increasingly recommended as smoking cessation aids. Therefore, the objectives of this study are to evaluate and compare the impact of e-cig (low- or high-power setting), HTP (IQOS system) and conventional cigarette emissions on the induction of oxidative stress, primary DNA damage, chromosomal aberrations and epigenetic lesions in a human bronchial epithelial cell model (BEAS-2B cell line).

## 2. Materials and Methods

### 2.1. E-Cig and Tobacco Products

The e-cig used in this study was the model “ModBox TC” (third-generation model) manufactured by NHOSS^®^ (Bondues, France). It was used with an “Air Tank” clearomizer equipped with a 0.5 Ω Kanthal coil and a partially closed air flow. Heating was activated one second before releasing the puff. The ModBox e-cig was tested at two power settings, 18 W (Mb-18W) and 30 W (Mb-30W), which corresponded to the lower- and upper-range power supplies recommended by the manufacturer for the coils used. The e-liquid used was a “blond tobacco” flavor (NHOSS^®^ brand) and contained propylene glycol <65%, glycerol <35%, food flavorings and nicotine at a concentration of 16 mg/mL. The HTP device used in this study was the IQOS 2.4 model manufactured by Philip Morris International (Neuchâtel, Switzerland), with IQOS heets (amber flavor from Philip Morris International) purchased from a local tobacco shop (Lille, France). The IQOS device was regularly cleaned every 20 heets, as recommended by the manufacturer. 3R4F research cigarettes were purchased from the University of Kentucky (Lexington, KY, USA).

### 2.2. Cell Culture

The adherent human bronchial epithelial BEAS-2B cell line (CLR-3588) was purchased from ATCC^®^ and was cultured as previously described [12]. For exposure experiments, cells were seeded onto Transwell-clear culture inserts with 0.4 µm pore size (Sigma Aldrich, Saint-Quentin Fallavier, France) pre-coated with 0.03 mg/mL type I collagen solution (Thermo Fisher Scientific, Courtaboeuf, France). Cells were kept submerged for 4 days in LHC-9 medium (Thermo Fisher Scientific), then an air–liquid interface (ALI) was established 24 h before the exposure by removing medium from the apical surface, exposing only the basal surface to medium.

### 2.3. Aerosol Generation and Cell Exposure

**Figure 1:**
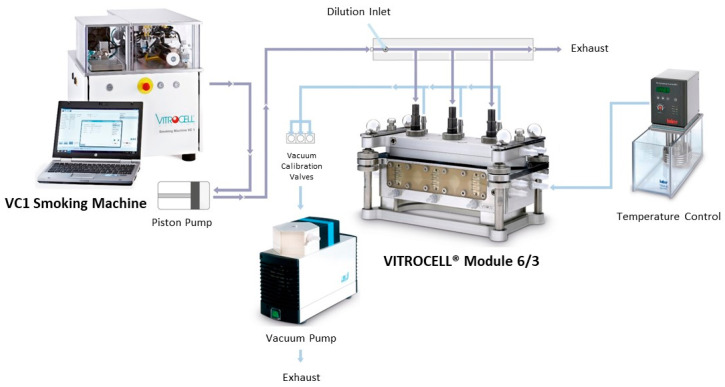
Vitrocell^®^ exposure system. VC1 smoking machine (for generation of e-cig, HTP and cigarette emissions) connected to a 6/3 exposure module allowing the direct exposure of cells cultured at the air–liquid interface on 6-well inserts.

The different aerosols were delivered using a Vitrocell^®^ System (Vitrocell, Waldkirch, Germany), as previously described [12,40] (Figure 1), except for the rate of emissions dilution. In the present study, aerosols were diluted in synthetic air consisting of 80% nitrogen and 20% oxygen (Linde, Noyal sur Vilaine, France), using a flow rate of 0.5 L/min. Cell culture inserts were transferred into the exposure module (Vitrocell 6/3 CF module) and exposed to different doses (defined in puff number) of the diluted HTP, e-cig or cigarette aerosol generated by the Vitrocell^®^ system. The negative control used in this study consisted of cells cultured at ALI and left in the incubator for the duration of exposure, as preliminary experiments showed that exposure to sterile air did not induce cytotoxicity, oxidative stress or inflammation and did not affect the transcriptome of BEAS-2B cells cultured at an ALI [40].

The Health Canada Intense (HCI) puff profile, which involved a 55 mL puff volume, 2 s puff duration and 30 s interpuff interval, was used for all products. For the 3R4F cigarette, all ventilation holes were blocked using adhesive tape during the experiments, in accordance with the recommendations for this smoking regime [41]. To ensure realistic experimental conditions and to avoid the generation of dry puffs, two regular e-cig users tested the e-cig using the puff duration and power settings as tested with the smoking machine. The users confirmed no dry puff sensation and sufficient aerosol production. In addition, the temperature of the generated aerosol can be considered as an indicator of experimental relevance and realism, according to the recommendations of French national organization and standardization [42]. The temperature of the aerosols generated from each device did not exceed 60 °C during all exposure periods [12], confirming the realism and the relevance of the exposures.

### 2.4. Intracellular ATP Assay

BEAS-2B cells were exposed to different puff numbers of diluted emissions (12, 60, 120, 240 and 360 puffs for HTP; 60, 120, 240 and 360 puffs for e-cig; 2, 4, 10, 20, 30 puffs for 3R4F cigarette). Cell viability was measured 24 h after exposure using the Cell Titer-Glo Luminescent Cell Viability assay kit (Promega, Charbonnières, France), as described previously [40]. Intracellular ATP was determined as percentages related to the ATP content in control cells arbitrarily set at a value of 100%, based on the number of puffs or nicotine content (mg) of each device’s emissions.

**Figure 2:**
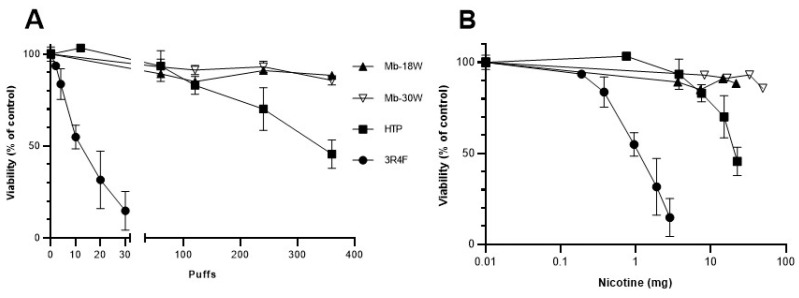
In vitro cytotoxicity of HTP, e-cig and cigarette emissions. BEAS-2B cells were exposed to an increasing number of puffs from HTP, e-cig (Mb-18W and Mb-30W) and 3R4F cigarette. Cell viability was evaluated by measuring the intracellular ATP level 24 h after exposure. Results are expressed as percentages relative to the ATP content in the unexposed cells, arbitrarily set at a value of 100%. Cell viability is expressed as a function of the number of puffs (**A**) or the nicotine content (mg) of each device’s emissions (**B**). Data represent the mean ± standard deviation (SD) of four independent culture replicates.

Nicotine content in the different emissions (under the same experimental conditions) was previously determined [12]. Briefly, nicotine was first transferred from 10 puffs of aerosols into two glass impingers with fritted nozzles placed in series containing methanol maintained at −40 °C. Quantification of nicotine in aerosol extracts was then performed by ultra-performance liquid chromatography as previously described [43]. Results showed that Mb-18W, Mb-30W, HTP and 3R4F cigarette tests delivered 60, 137, 63 and 95 µg of nicotine/puff, respectively, under the HCI puffing profile [12]. Finally, nicotine content indicated on the abscissa of the cytotoxicity figure (Figure 2B) was determined by the nicotine concentration per puff (depending on the device) multiplied by the number of puffs (depending on the exposure duration).

### 2.5. Measure of the Nuclear Activity of the Nuclear Factor Erythroid 2-Related Factor 2 (Nrf2)

Nuclear extracts were first collected using the Nuclear Extract Kit (Active Motif, Carlsbad, CA, USA). Transcription factor DNA-binding activity was then studied using the TransAM^®^ Nrf2 kit (Active Motif), according to the manufacturer’s recommendations. Briefly, nuclear proteins (10 μg) were added to wells containing consensus Nrf2-binding site element oligonucleotides and incubated for 2 h at room temperature. Spectrophotometric quantification was accomplished after the addition of secondary antibody conjugated to horseradish peroxidase. The binding activity values were presented relative to activity measured in the negative control.

### 2.6. Gene Expression Analysis

Total RNAs were extracted using the RNeasy Mini Kit (Qiagen, Courtaboeuf, France) on the QIAcube automaton, following the manufacturer’s instructions. Expression of target genes was measured by quantitative real-time PCR of corresponding reverse-transcribed mRNAs. One microgram of total RNAs was reverse-transcribed into cDNAs using the High-Capacity cDNA Reverse Transcription kit (Applied Biosystems, Waltham, MA, USA). qPCRs were carried out with the StepOnePlus thermocycler (Applied Biosystems), using the TaqMan Fast Advanced Master Mix (Applied Biosystems) and the following TaqMan Assays: Hs01110250_m1 for *HMOX1*, Hs01045993_g1 for *NQO1* and Hs99999901_s1 for *18S*. Amplification curves were read with StepOne software V2.1 using the comparative cycle threshold method. The relative quantification of the steady-state mRNA levels was normalized against *18S* RNA. Results are expressed as the fold-change relative to the levels in control cells arbitrarily set at a value of 1.

### 2.7. Western Blot Analyses

Total cellular protein extracts were obtained by way of cell lysis. Twenty micrograms of protein underwent electrophoresis, and immunoblotting was performed with mouse monoclonal anti-human HO-1/HMOX1 (1:1000, Santa Cruz Biotechnology, Dallas, TX, USA, sc-136960), mouse monoclonal anti-human NQO-1 (1:1000, Novus Biologicals, Littleton, CO, USA, NB200-209) and mouse monoclonal anti-human β-actin (1:50,000, R&D Systems Inc., Minneapolis, MN, USA, MAB8929).

### 2.8. Comet Assay

The comet assay was performed under alkaline conditions (pH >13) with or without the presence of hOGG1 (to detect specifically oxidative DNA damage) in compliance with previously described protocols [44,45,46,47]. A positive control was carried out in parallel with cells treated for 3 h with methyl methanesulfonate (MMS) (15 µg/mL). After exposure, cells were washed with PBS and immediately harvested by trypsinization. Cell viability was assessed using the Trypan Blue assay. To exclude cytotoxicity as a confounding factor, inserts showing more than 70% relative cell viability, as recommended by Tice et al. [46], were then immediately submitted to the comet assay. For each condition, 2 independent exposures (i.e., 2 inserts) were realized and 4 technical replicates were carried out for each insert (i.e., 2 slides with hOGG1 and 2 slides without hOGG1). One hundred randomly selected cells per slide were then blindly examined (i.e., four hundred cells per condition). The level of primary DNA damage was expressed as the percentage of DNA in the tail (% tail intensity).

### 2.9. Micronucleus Test

The micronucleus test was performed in compliance with our previously described protocol [44]. Briefly, at the end of each exposure, cells were washed, trypsinized, reseeded in a new 6-well plate and then incubated at 37 °C for an additional 2-day recovery period (i.e., 48 h). A positive control of Mitomycin C (0.1 µg/mL) was included (3 h of treatment). For each condition, 3 independent exposures (i.e., 3 inserts) were realized and duplicate technical replicates per insert were prepared for analysis (i.e., 6 slides in total per condition). Slides were independently coded and analyzed. Micronuclei (MN) were identified according to recommended criteria [48,49] and scored in 1000 intact mononucleated cells per slide (i.e., 6000 mononucleated cells/condition). Each treatment was coupled with an assessment of cytotoxicity evaluated by calculating the percentage of relative population doubling (RPD). According to the OECD 487 guideline [50], concentrations that led to a relative population doubling greater than 45 ± 5% were considered to have low or no cytotoxicity.

### 2.10. Global DNA Methylation

The global DNA was extracted using the Qiacube automaton (Qiagen, Redwood City, CA, USA) following the instructions of the QiaAmp DNA mini kit (Qiagen). Then, the quantification of methylated DNA was carried out by the 5-mC DNA ELISA Kit (Zymo Research, Irvine, CA, USA). Levels of 5-methylcytosine (5-mC) were determined using an indirect ELISA technique, where denatured single-stranded DNA samples (100 ng) were applied to well surfaces coated with 5-mC-binding buffer. Wells were first incubated with a monoclonal anti-5-mC antibody and then with the secondary antibody conjugated with horseradish peroxidase. Detection of 5-mC occurred after the addition of the horseradish peroxidase developer. The colorimetric readout was quantifiable by spectrophotometry using a microplate reader at 405–450 nm. Percent 5-mC in a DNA sample was accurately quantified from a standard curve generated with specially designed controls included with the kit. 

### 2.11. ELISA Assay to Detect Histone Modifications

The EpiQuik^TM^ Total Histone Extraction kit (Epigentek, Farmingdale, NY, USA) was used according to the manufacturer to obtain acid extracts containing histones. Different histone modifications were then measured using the following EpiQuik^TM^ Global Histone Quantification kits (Epigentek): acetylation of lysine 9 of histone H3 (H3K9ac), tri-methylation of lysine 4 of histone H3 (H3K4me3), tri-methylation of lysine 9 of histone H3 (H3K9me3) and tri-methylation of lysine 27 of histone H3 (H3K27me3). The total histone H3 was assessed in order to standardize the results. Total or modified histone H3 was captured in wells coated with anti-histone H3 antibody or one of the modifications. The captured H3 histones could then be detected with a specific detection antibody for each modification, followed by a fluorescence development reagent (fluorescent secondary antibody). The amount of histone was proportional to the fluorescence intensity obtained with a wavelength of 530ex/590em nm.

### 2.12. Statistical Analyses

Statistical analyses were performed using the non-parametric Mann–Whitney test, except for the micronucleus test for which Fisher’s exact test was used, as recommended by Matsushima et al. [51]. Data were considered significantly different when *p* < 0.05. Data analyses and graphs were made with PRISM 8.0.2 software (Graph Pad, San Diego, CA, USA).

## 3. Results

### 3.1. Evaluation of the Cytotoxicity after Exposure to E-Cig or Tobacco Product Emissions

In order to compare the cytotoxicity of the different devices, BEAS-2B cells were exposed to a wide range of doses of HTP, e-cig (Modbox set to 18 or 30 W) or 3R4F cigarette emissions. A cell viability assay was performed 24 h after exposure by measuring the intracellular ATP content, which is directly proportional to the number of living cells. HTP emissions caused a decrease in ATP concentration when the number of puffs increased from 120 puffs (83% viability); ATP concentration reached 45% of cell viability after exposure to 360 puffs (Figure 2A). Cytotoxicity was more pronounced for 3R4F cigarette emissions, which caused a 45% decrease in cell viability after 10 puffs. Viability reached 15% after exposure to only 30 puffs of CS. By contrast, no significant decrease in cell viability was observed for up to 360 puffs of e-cig, both at low (18 W)- and high (30 W)-power settings. When the cell viability after the different emissions was expressed as a function of the amount of nicotine (Figure 2B), the ranking of devices based on their cytotoxicity remained similar: HTP was less cytotoxic than conventional cigarettes whereas the e-cig was not cytotoxic. The EC_50_ (dose decreasing to 50% cell viability) was 12 puffs or 0.87 mg of nicotine for the 3R4F cigarette whereas it was 350 puffs or 22 mg of nicotine for the HTP. Doses not overly toxic (i.e., >85% of cell viability) within the 24 h exposure were chosen for further analyses of oxidative stress: 60 and 120 puffs for the e-cig and HTP, and 2 and 4 puffs for the 3R4F cigarette.

### 3.2. Study of Oxidative Stress after Exposure to E-Cig or Tobacco Product Emissions

**Figure 3:**
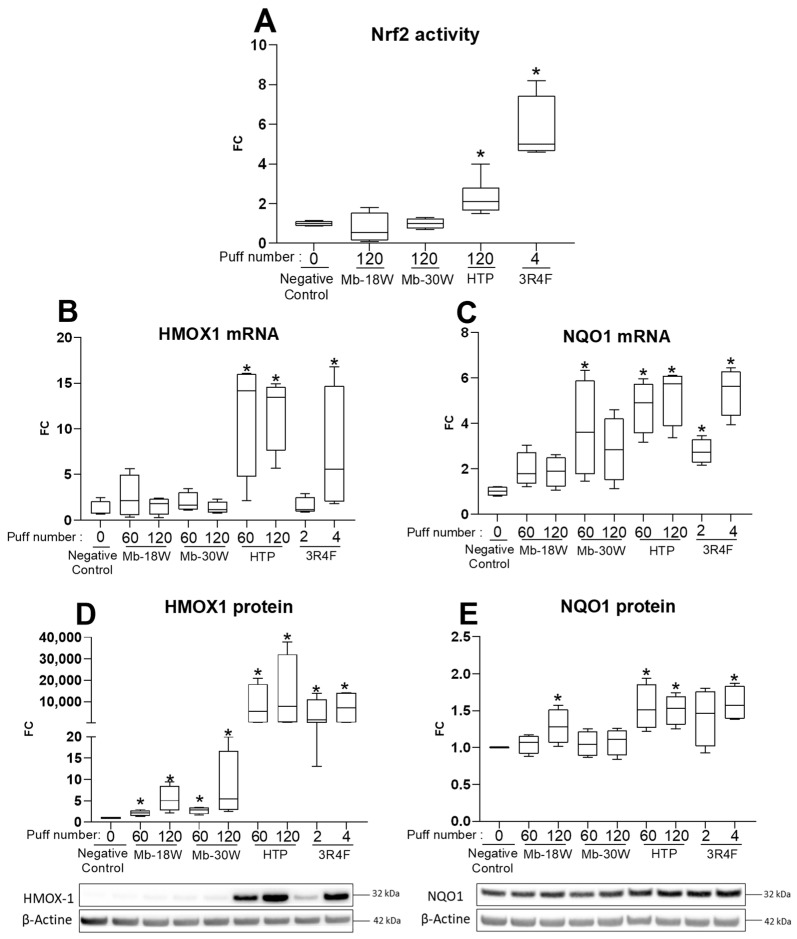
Evaluation of the Nrf2 antioxidant stress response in BEAS-2B cells exposed to HTP, e-cig and cigarette emissions. Cells were exposed to 60 or 120 puffs of HTP and e-cig (Mb-18 W and Mb-30W), or to 2 or 4 puffs of 3R4F cigarette. (**A**) Nuclear activity of Nrf2 was evaluated 4 h after exposure. Results are expressed as fold-change (FC) relative to Nrf2 activity in unexposed cells (negative control) arbitrarily set at value of 1. (**B**,**C**) Expression of HMOX1 and NQO1 mRNA was evaluated by RTqPCR 24 h after exposure. Results are expressed as fold-change (FC) relative to control cells, arbitrarily set at a value of 1. (**D**,**E**) Expression of HMOX1 and NQO1 proteins was evaluated 24 h after exposure by Western Blotting using appropriate antibodies and developed with an electrochemiluminescence reagent. Results are reported by representative Western blot images and relative densitometric bar graphs obtained after normalization with β-Actin expression. Results are expressed as fold-change (FC) relative to control cells, arbitrarily set at a value of 1. Data represent the median and interquartile range from four independent culture replicates. * *p* < 0.05 compared with negative control cells.

The study of oxidative stress was first assessed by measuring the nuclear activity of the nuclear factor erythroid 2-related factor 2 (Nrf2) in response to e-cig, HTP or cigarette exposures. Indeed, the activation of Nrf2 is an efficient antioxidant defensive mechanism used by cells to counteract oxidative stress. The activation of Nrf2 was tested 4 h after exposure of BEAS-2B cells to the different emissions (Figure 3A). Both HTP (120 puffs) and cigarette (4 puffs) emissions significantly increased nuclear Nrf2 DNA-binding activity compared with negative control cells. By contrast, Mb-18W and Mb-30W (120 puffs) had no effect on this transcription factor’s DNA-binding activity. Expression of the downstream targets of the Nrf2 transcription factor was then analyzed 24 h after cell exposure: the mRNA and protein levels of HMOX1 and NQO1 were measured by RT-qPCR and by Western blotting, respectively. Exposure to HTP (60 or 120 puffs) and 3R4F cigarette (4 puffs) emissions induced a strong increase in HMOX1 mRNA (Figure 3B). HMOX1 was also strongly enhanced at the protein level by both tobacco products, while the expression of the HMOX1 protein was increased to a smaller extent by e-cig (Figure 3D). In addition, 60 or 120 puffs of HTP and 4 puffs of the cigarette induced the expression of NQO1 in a similar manner, with an approximately 5-fold increase at the mRNA level (Figure 3C) and a 1.5-fold increase at the protein level (Figure 3E). A weak (but statistically significant) induction of NQO1 protein expression (Fold-change = 1.3) was also shown after exposure to 120 puffs of Mb-18W. However, this induction was not observed with the Mb-30W condition. In conclusion, the study of the activation of the Nrf2 pathway by tobacco and vaping products showed that, unlike e-cig, HTP induced an antioxidant response at a level comparable to that observed with conventional cigarettes, even though doses of tobacco cigarettes were much lower (4 puffs) than those of HTP (120 puffs) and achieved the same effect.

### 3.3. Assessment of Genotoxicity and Mutagenicity after Exposure to E-Cig or Tobacco Product Emissions

**Figure 4:**
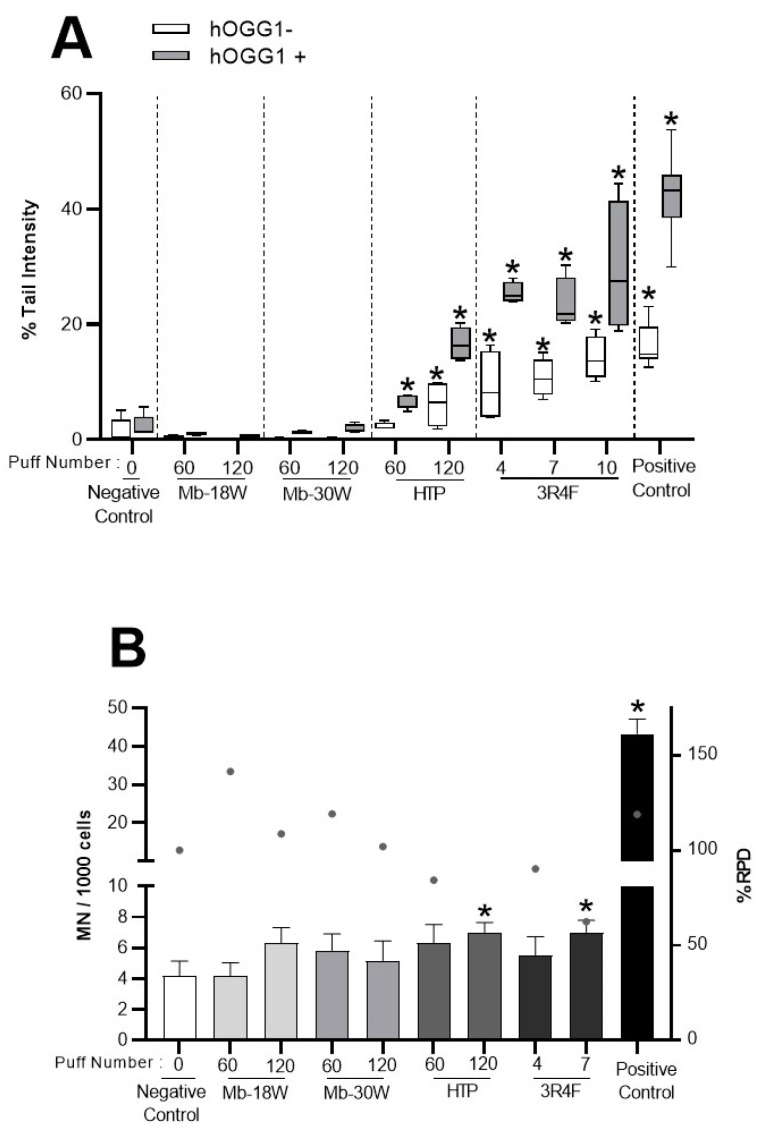
Measure of DNA damage and chromosomal aberrations in BEAS-2B cells exposed to HTP, e-cig and cigarette emissions. Cells were exposed to 60 or 120 puffs of HTP and e-cig (Mb-18W and Mb-30W) or to 4, 7 or 10 puffs of 3R4F cigarette. (**A**) DNA damage was measured by the comet assay under alkaline conditions in the presence (grey) or absence (white) of hOGG1. Cells were also treated for 3 h with MMS (15 μg/mL) as positive control. Results are expressed as % of DNA in the tail of the comet. Data represent the median and interquartile range from 4 technical replicates resulting from two independent culture replicates (with 100 nuclei analyzed per replicates). * *p* < 0.05 compared with negative control cells. (**B**) Chromosomal aberrations were measured by the micronucleus test. Cells were treated for 3 h with Mitomycin C (0.2 μg/mL) as positive control. Results are expressed as the number of micronucleated cells (MN) per 1000 cells. The % relative population doubling (RPD) is used as a measure of cytotoxicity. Data represent the mean ± SD of three independent culture replicates. * *p* < 0.05 compared with negative control cells.

The generation of oxidative stress has been proposed as the underlying mechanism involved in the genotoxicity of many toxic compounds [52,53]. Primary DNA damage was first assessed after e-cig and tobacco product exposure using the comet assay. BEAS-2B cells were exposed to 60 or 120 puffs of HTP or e-cig (Mb-18W and Mb-30W) or 4 to 10 puffs of CS. DNA strand breaks were evaluated using the alkaline comet assay. In addition, the comet assay modified using the hOGG1 repair endonuclease was used to specifically detect oxidative lesions. Primary DNA damage is expressed as the percentage of DNA in the comet tail and is shown in Figure 4A. In the standard comet assay, 3R4F cigarette exposure induced a statistically significant, dose-dependent increase in DNA breaks (up to 13.6% tail intensity at higher dose, vs. 0.2% for the negative control). A statistically significant increase in DNA damage was also observed after exposure to 120 puffs of HTP. With the hOGG1-modified comet assay, statistically significant increases in tail intensity were observed for both 3R4F cigarette and HTP emissions at all doses tested (notably 27.5% for 10 puffs of CS and 16.3% for 120 puffs of HTP, vs. 1.5% for the negative control). On the contrary, under the two experimental conditions tested (i.e., with or without hOGG1), e-cig exposure did not induce any biologically nor statistically significant increases in DNA strand breaks or oxidative DNA damage, regardless of the dose (i.e., 60 and 120 puffs) or the power of e-cig (i.e., 18 and 30 W).

In addition, the in vitro micronucleus test was used to assess the induction of chromosomal breakage and aneuploidy 48 h after exposure of BEAS-2B cells to the different emissions (Figure 4B). Cells exposed to 10 puffs of the 3R4F cigarette were ultimately not analyzed due to excessive cytotoxicity (i.e., <45% viability compared with negative control). Only a slight statistically significant increase in the number of micronuclei was shown in cells exposed to 120 puffs of HTP and 7 puffs of CS (7 and 7 micronuclei vs. 4.2 micronuclei for 1000 cells in negative control, respectively). No significant induction of micronucleus formation was observed in BEAS-2B cells exposed to e-cig (Mb-18W and Mb-30W). Taken together, these data suggested that, under these experimental conditions in BEAS-2B cells, both tobacco products (HTP and conventional cigarettes) induced primary DNA damage and chromosomal aberrations. By contrast, e-cig (independently of the power tested) did not cause genetic damage.

### 3.4. Study of Impact of E-Cig or Tobacco Product Emissions in Global DNA Methylation and Histone Modulations

**Figure 5:**
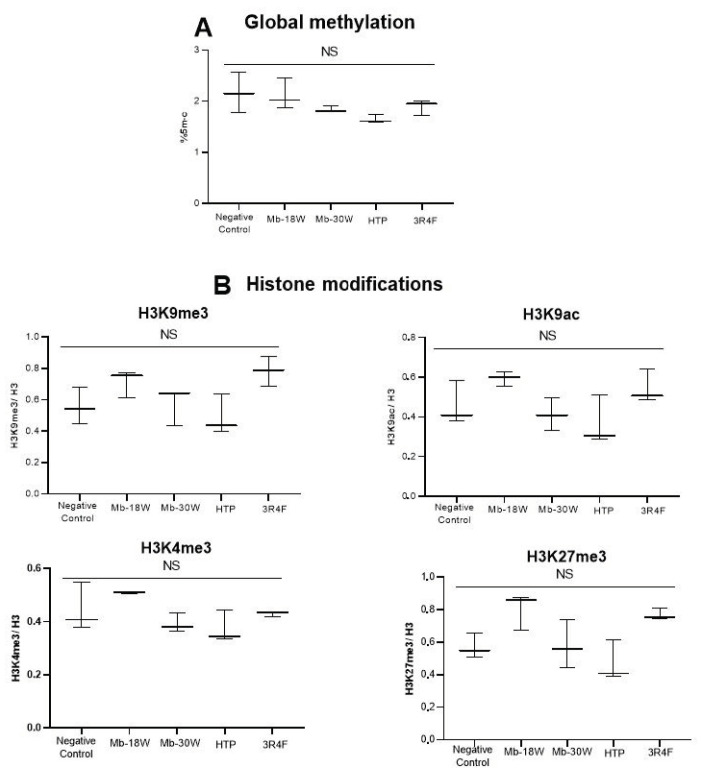
Evaluation of global DNA methylation and histone modulations in BEAS-2B cells exposed to HTP, e-cig and cigarette emissions. Cells were exposed to 120 puffs of HTP and e-cig (Mb-18W and Mb-30W) or to 4 puffs of 3R4F cigarette. (**A**) Global DNA methylation was measured 24 h after exposure. Results are expressed as percentages of global methylation. (**B**) Modifications of histones (H3K9me3, H3K9ac, H3K4me3 and H3K27me3) were measured by ELISA 24 h after exposures. Results are expressed as the ratio of modified histone/total H3. Data represent the median from three independent culture replicates. NS: not significant.

In order to compare the epigenetic impact of e-cig, HTP and cigarettes, the global DNA methylation and the post-translational modifications of histone H3 were investigated in BEAS-2B cells exposed to 120 puffs of HTP and e-cig (Mb-18W or Mb-30W) or to 4 puffs of a 3R4F cigarette. These different exposures did not induce statistically significant modulations of global DNA methylation (Figure 5A) in comparison with unexposed cells. Similarly, no modulation of histones H3K9me3, H3K9ac, H3K4me3 and H3K27me3 was demonstrated after acute exposure to the different emissions in the BEAS-2B cell line (Figure 5B).

## 4. Discussion

Both e-cig and HTP provide alternatives for smokers and are increasingly used as smoking cessation aids, while the risk reduction effects of these new products are still the subject of controversial debate in humans [54,55]. In this context, our study was implemented with the objective to compare the effects of e-cig, HTP and cigarette emissions on the induction of oxidative stress and genetic and epigenetic lesions in human bronchial epithelial cells. We first report that HTP were less cytotoxic than conventional cigarettes whereas the e-cig used was not cytotoxic in BEAS-2B cells. The number of puffs required to reach EC_50_ was about 29-fold higher for the HTP emissions (350 puffs) compared with CS exposure (12 puffs). These data were consistent with those of Chapman et al. who found a 38-fold difference between EC_50_ of both devices in the same cell line [37]. On the other hand, we did not observe any significant decrease in cell viability after exposure of BEAS-2B cells to e-cig, regardless of the power used (18 or 30 W). Escobar et al. also compared the impact of the wattages (40 and 85 W) of a third-generation e-cig device on cytotoxicity in 16HBE airway epithelial cells exposed to different aerosolized mixtures of propylene glycol and glycerol. A modest increase in LDH release (<10% of the positive control) was seen only with propylene glycol aerosolized using the e-cig at high power (85 W) [56]. By contrast, other authors have demonstrated a clear dose-dependent cytotoxicity after exposure to e-cig aerosols but only under specific or extreme conditions with an airflow vent closed [57] or with e-liquid containing vitamin E acetate [58].

Consistent with results of the cytotoxicity assessment, an antioxidant response was only observed after exposure of BEAS-2B cells to HTP or CS. Indeed, both tobacco products increased the binding activity of Nrf2 to antioxidant response element (ARE) and the expression of its downstream targets HMOX1 and NQO1. These results were in accordance with data from Giebe et al. who showed that stimulation of primary human endothelial cells with aqueous extracts of HTP and cigarettes led to activation of the Nrf2 antioxidant defense system [59]. In this latter study, as in ours, the doses required to induce oxidative stress were much lower for cigarettes than for HTP. In addition, consistently in both studies, e-cig had no significant effect on the Nrf2 pathway. By contrast, other studies have shown that e-cig used in specific conditions induced a Nrf2-dependent oxidative stress. Indeed, an increase in HMOX1 and NQO1 gene expression and carbonylated proteins was observed in the 16HBE cell line exposed to aerosolized glycerol when an e-cig device was used at high power of 85 W while no effect was observed on antioxidant response genes after an exposure at 40 W power [56]. Moreover, Canchola et al. reported that aerosol generated from an e-liquid containing vitamin E acetate resulted in up-regulation of NQO1 and HMOX1 genes in BEAS-2B cells, due to thermal decomposition products of vitamin E acetate [58].

The induction of the antioxidant stress response by tobacco products was also correlated with generation of oxidative DNA damage (measured by the hOGG1-modified comet assay) and DNA strand breaks in BEAS-2B cells exposed to HTP and CS. In addition, assessment of chromosomal damage showed that the micronuclei frequency was statistically significantly increased after exposure to 3R4F cigarettes and HTP, evidencing the genotoxic activity (i.e., breakage and aneuploidy) of these two tobacco products under our experimental conditions. These latest results are consistent with those of the comet assay, demonstrating that the primary DNA damage observed can lead to chromosomal aberrations after cell division. An in vivo study has previously shown that exposure to HTP caused oxidative damage in the lungs and also DNA strand breaks in the leucocytes of exposed rats [38]. Other authors have also observed an increase in micronuclei frequency after HTP exposure [37,39], while Thorne et al. concluded with negative results for a micronucleus test performed with total particulate matter from HTP in the Chinese hamster V79 cell line [34].

For the study of e-cig genotoxicity, we showed that e-cig did not induce primary DNA lesions (no DNA breaks or oxidative DNA damage), or chromosomal aberrations, in BEAS-2B cells up to an exposure of 120 puffs. Contradictory conclusions have often been published about the genotoxic activity of e-cig. The lack of genotoxic potential of e-cig under our experimental conditions is consistent with the study of Tellez et al. who evaluated the genotoxicity of aerosols generated from 10 flavored e-liquids with or without nicotine in 3 different immortalized oral epithelial cell lines. None of aerosols increased DNA damage (assessed by the alkaline comet assay), unlike the CS that caused a dose-dependent increase in DNA strand breaks in the three cell lines [31]. Similar to our work, negative results were also reported in several studies performing the micronucleus test after e-cig exposure [3,32,33]. On the contrary, other in vitro studies have shown a positive response to the comet assay [29,30,60,61] and/or to the micronucleus test [30,31] after e-cig exposure. These positive results could be explained by specific conditions used for e-cig exposure. Indeed, an exposure to 20 puffs performed with a short interpuff interval (20 s) induced DNA lesions in immortalized human alveolar A549 cells [60]. In another study using normal epithelial cells and head and neck squamous cell carcinoma cell lines, repeated exposure to e-cig aerosol extracts for 1 week and 2 months resulted in an increase in comet tail length and an accumulation of gamma-H2AX foci, independently of the nicotine present in the e-liquid [29]. Al-Saleh et al. assessed the genotoxicity of 33 e-liquids in human lymphoblastoid TK6 cells using the comet assay. All the e-liquids induced DNA damage in these cells, with a greater genotoxic effect in the presence of metabolic activation [30]. However, these last two studies used immerged cells with e-liquids or e-cig aerosol extracts diluted in culture media. This mode of cell exposure appears less relevant than air–liquid interface-cultured cells exposed to aerosols generated via a smoking machine [62].

The conflicting data published in the literature about the cytotoxicity, oxidative stress generation and genotoxicity of e-cig and HTP can be explained by several factors, such as models of e-cig or HTP, the type of product tested (e.g., whole smoke aerosol, smoke condensate or extract, particulate/gas phase, e-liquid itself, etc.), e-liquid composition, usage patterns (e.g., power of e-cig), cell models (e.g., lung epithelial cells, oral epithelial cells, oropharynx cells, ”regulatory” cells, rodent vs. human origin, p53 status, antioxidant capacities, etc.), mode of cell exposure (e.g., submerged cells or ALI condition), various endpoints and durations of exposure (acute or repeated). Another parameter that varied between different studies was the puff regime. Indeed, diverse standardized smoking or vaping regimes have been created on the basis of users’ puffing behavior (or topography) which varied greatly between e-cig, HTP and cigarettes. Thus, the ISO 20768:2018 puff regime was recommended for e-cig, whereas HCI and ISO3308:2012 profiles are currently used for tobacco products. In the present study, the use of the HCI regime represented a limitation for the study of e-cig. However, we chose to use a single puff regime for all exposures in order to compare the toxicity of the different devices under the same laboratory conditions. In addition, we assessed the toxicity endpoints on human p53-competent BEAS-2B cells after an acute exposure to e-cig, HTP or 3R4F aerosols. Prolonged exposures to aerosols could reveal secondary or cumulative toxicological effects in lung cells. We have previously demonstrated, using an in vivo murine model, that a chronic exposure (6 months) to 3R4F cigarettes and to Mb-30W e-cig induced oxidative DNA damage in the lungs and the liver [63]. By contrast, Mb-18W e-cig did not induce in vivo primary DNA lesions. In addition, no chromosomal aberrations or gene mutations were evidenced in this murine model regardless of the type of product. It will be interesting to assess the genotoxicity and mutagenicity of HTP in animals chronically exposed to their emissions, given that HTP have already caused DNA lesions after acute exposure of the BEAS-2B cells. Repeated induction of DNA damage due to long-term exposure, combined with a dysfunction in DNA repair pathways, could generate accumulated mutations and promote carcinogenesis. Reiterated exposures (in animal or cell models) should also help to identify epigenetic alterations. Indeed, neither histone modulations nor global DNA methylation changes were found in our in vitro model after acute exposure, regardless of the type of emissions (including CS). However, global hypomethylation has already been observed in BEAS-2B cells after repeated daily exposures to CS [26]. Similarly, Wang et al. showed that a reiterated exposure to CS enhanced the methylation of histone H3 (H3K27me3) in a time-dependent manner in human bronchial epithelial cells [25]. Thus, repeated exposures appear to be more relevant to demonstrate epigenetic alterations and, therefore, studies in cell and animal models chronically exposed to e-cig and HTP are urgently required.

The potential for cytotoxicity, oxidative stress induction and genotoxicity may be associated with the chemical constituents of emissions and the production of carbonyl compounds, PAHs, volatile organic compounds, heavy metals, etc. In a previous study [12], we compared the chemical composition of the different emissions used in the present study. The differences in the in vitro toxicity between the devices are consistent with the concentrations of chemical compounds measured previously. Indeed, we shown that HTP emitted less PAHs and carbonyls than conventional cigarettes. However, amounts of these compounds in HTP aerosols were still higher than in e-cig aerosols. The presence of higher concentrations of aldehydes, PAHs, quinones, ketones and free radical species in the emissions of tobacco products [64] may contribute to enhanced oxidative stress in exposed cells. Among these compounds, formaldehyde, acetaldehyde and benzo-[a]-pyrene are well-known DNA-damaging agents and their presence at high concentrations may explain the generation of oxidative DNA lesions and/or DNA strand breaks in HTP- and CS-exposed BEAS-2B cells. In contrast, the concentrations of carbonyl compounds and PAHs measured in the emissions of e-cig were sufficiently low, due to the absence of combustion during e-cig use, which may explain the lack of cytotoxicity, Nrf2 antioxidant response and genotoxicity in BEAS-2B cells exposed to e-cig.

In conclusion, this study, which compared e-cig, HTP and cigarettes under the same experimental conditions, provides important and innovative results for health risk assessment, as it allowed ranking the devices according to their in vitro toxicity. Results reveal that HTP, unlike e-cig, elicit a biological response very similar to that of CS, but only after a more intensive exposure: both tobacco products induce cytotoxicity, Nrf2-dependent oxidative stress and genetic lesions in human epithelial pulmonary cells. Therefore, the health risk of HTP should not be underestimated. Animal studies should follow this work in order to determine the long-term toxicity and the tumorigenic potential of these emerging tobacco and vaping products.

## Data Availability

Not applicable.

## Abbreviations

**ALI**	air–liquid interface
**ARE**	antioxidant response element
**CS**	cigarette smoke
**EC_50_**	dose decreasing to 50% cell viability
**e-cig**	electronic cigarettes
**HTP**	heated tobacco products
**Mb-18W**	Modbox e-cig model set at 18 W
**Mb-30W**	Modbox e-cig model set at 30 W
**Nrf2**	Nuclear factor erythroid 2-related factor 2
**PAHs**	polycyclic aromatic hydrocarbons
